# *JARID1A*, *JMY*, and *PTGER4* Polymorphisms Are Related to Ankylosing Spondylitis in Chinese Han Patients: A Case-Control Study

**DOI:** 10.1371/journal.pone.0074794

**Published:** 2013-09-19

**Authors:** Wei Chai, Zijian Lian, Chao Chen, Jingyi Liu, Lewis L. Shi, Yan Wang

**Affiliations:** 1 Department of Orthopaedics, Chinese People’s Liberation Army General Hospital, Beijing, China; 2 Department of Orthopaedics, Tianjin Hospital, Tianjin, China; 3 Medical School of Nankai University, Tianjin, China; 4 Department of Orthopaedics, University of Chicago Hospital, Chicago, Illinois, United States of America; University of Birmingham, United Kingdom

## Abstract

Susceptibility to ankylosing spondylitis (AS) is largely genetically determined. *JARID1A*, *JMY* and *PTGER4* have recently been found to be associated with AS in patients of western European descent. We aim to examine the influence of *JARID1A*, *JMY*, and *PTGER4* polymorphisms on the susceptibility to and the severity of ankylosing spondylitis in Chinese ethnic majority Han population. This work can lead the clinical doctors to intervene earlier. Blood samples were drawn from 396 AS patients and 404 unrelated healthy controls. Both the AS patients and the controls are Han Chinese. The AS patients are classified based on the severity of the disease. Thirteen tag single nucleotide polymorphisms (tagSNPs) in *JARID1A*, *JMY* and *PTGER4* are selected and genotyped. Frequencies of different genotypes and alleles are analyzed among the different severity AS patients and the controls. The rs2284336 SNP in *JARID1A*, the rs16876619 and rs16876657 SNPs in *JMY* are associated with susceptibility of AS. The rs11062357 SNP in *JARID1A*, the rs2607142 SNP in *JMY* and rs10440635 in *PTGER4* are related to severity of AS. Haplotype analyses indicate *PTGER4* is related to susceptibility to AS; *JARID1A* and *JMY* are related to severity of AS.

## Introduction

Ankylosing spondylitis (AS) is a chronic inflammatory disease characterized by inflammation in the sacroiliac joints and spine which causes joint and bone erosion and even ankylosis [1]. Most AS patients develop first symptoms before they are 30 years old [2]. Radiographic progression during the first 10 years of disease is an important prognostic indicator of disease severity; some recent studies suggest early presentation of structural damage can be considered as a good predictor of further damage [3-5].

The severity of AS is largely genetically determined. Most genetic studies focus on disease susceptibility; however, the published literature lacks candidate gene association studies for disease severity of AS [6]. The goal of this study is to examine several previously identified gene polymorphisms, and their influence on susceptibility to AS and severity of disease, all with the intention of earlier intervention leading to better outcome.

The genes studied in the present report include *JARID1A, JMY*, and *PTGER4*. *JARID1A* (jumonji, AT-rich interactive domain 1A) is also known as *KDM5A* (lysine-specific demethylase 5A) and RBP2 (retinoblastoma-binding protein 2) [7]. *JARID1A* encodes the JARID1A protein and regulates gene expression involved in numerous cellular functions, including tumorigenesis. JARID1A increases H3K4me3 (tri-methylated histone H3 at Lysine 4) which is recognized by plant homeodomain (PHD), leading to the altered programs of gene expression and progression of tumor [8]. The dysregulation of PHD finger has been implicated in a variety of human diseases including immune disorders and cancer [9]. Additionally, JARID1A interacts with estrogen receptor alpha, and much published literature has studied the relationship between JARID1A and breast cancer [8,10,11].

*JMY* encodes for JMY (junction-mediating and regulatory protein), This protein is a transcription co-factor. It was originally identified as a p300-binding protein, and it can augments the p53 tumor suppressor response [12]. *PTGER4* encodes EP4R (prostaglandin E receptor 4) which is the antagonism to inhibit cell growth, proliferation, and metastasis of breast cancer cells; in particular, *PTGER4* regulates the aggressive phenotypes of inflammatory breast cancer cells [13]. Thus, higher level of *PTGER4* and EP4R may lead tumor cell to proliferate. A recent study supports the hypothesis that PTGER4 receptor antagonists may be an alternative approach to prevent tumor metastasis [14].

In genome-wide association studies, rs11062385 in *JARID1A*, rs16876657 in *JMY*, and rs10440635 in *PTGER4* are related to AS susceptibility in patients of western European descent [15-17]. We hypothesize that the relationship between *JARID1A, JMY, PTGER4* and AS exists in other populations; additionally, particular single nucleotide polymorphisms (SNPs) of these genes may predict the severity of AS. This is a replication study, Chinese Han population is more than one billion. It is reasonable to support the western results using Chinese Han population. In this study, we examined *JARID1A, JMY*, and *PTGER4* genes in patients of the Chinese ethnic majority Han population.

## Methods

### 1: Study population

In this study, 396 AS patients and 404 unrelated healthy controls who are age and sex-matched are recruited. All AS patients and normal controls are Han Chinese. The Han ethnic group makes up 92% of the population in China and 20% of the global population, making it the largest ethnic group in the world. These samples are collected from PLA general hospital from 2010 to 2013. All AS patients are HLA-B27 positive and they are treated by non-steroidal anti-inflammatory drug routinely; no other treatments are used for patients. In the patient group, 354 male (89.4%) and 42 female (10.6%) are recruited; the average age is 29.6 years (range 16 to 60 years) (Table 1). In the control group, 370 male (91.6%) and 34 female (8.4%) are recruited; the average age is 30.0 years (range 16 to 60 years). Neither sex nor age distributions show significant differences between AS patients and control (p=0.291, 0.670 respectively). The average duration since AS diagnosis is 11.5 years (range 8 to 18 years). The diagnosis of AS has been made by experienced rheumatologists according to the modified New York criteria [18]. The diagnosis was reconfirmed by different rheumatologists. The diagnosis was made before the genetic information was genotyped. In another word, the phenotype was recorded by the rheumatologists blinded to the genetic information. Subjects with inflammatory bowel disease, psoriasis, rheumatoid arthritis, or other autoimmune diseases are excluded from both the AS and the control group.

**Table 1:** Demographic data of AS patients and controls.

		Cases (396)	Controls (404)	p-value
Sex	male	354 (89.4%)	370 (91.6%)	0.291
	female	42 (10.6%)	34 (8.4%)	
Age		29.6±8.5	30.0±9.4	0.670
Duration of diagnosis		11.5±2.1	N/A	
BASFI		3.94±1.45	N/A	
BASDAI		5.60±1.25	N/A	
mSASSS		13.7±15.0	N/A	

There is no significant difference in age and sex-distribution between AS patients and controls. Numerical values presented as mean±standard deviation. BASFI : Bath ankylosing spondylitis function index. BASDAI: Bath ankylosing spondylitis disease activity index. mSASSS: modified Stokes ankylosing spondylitis Spine Score.

### 2: Basic data acquisition

The Bath AS function index (BASFI) and Bath AS disease activity index (BASDAI) are administered to the patients using questionnaires; these indexes are the most widely used tools for the assessment of AS functional status and activity [19,20]. The modified Stokes AS Spine Score (mSASSS) is a validated scoring system for spinal structural changes [21]. The lateral views of standard radiographs of the cervical and lumbar spine are used to derive a mSASSS score for each patient [22,23]. Three of the authors separately assigned the mSASSS scores, and we used the average.

### 3: Severity classification

How to classify AS severity is still a field of discussion[24]. . In this study, we define severe type of AS as the disease form in those patients within first ten years of diagnosis who satisfied the indications of surgery, which include inability to stand upright, inability to look straight ahead, or compression of the viscera due to kyphosis that manifests as pain [25]. Patients with the normal type of AS exhibit inflammation of sacroiliac joints, but their spine and other joints were relatively spared; these patients have required only medical treatment. By this definition, 82 AS patients were the severe type, and 314 AS patients were the normal type (Table 2).

**Table 2:** Clinical features comparing severe AS and normal AS.

		severe AS (82)	normal AS (314)	p value
Sex	male	76 (92.7%)	276 (87.9%)	0.220
	female	6 (7.3%)	38 (12.1%)	
Age		31.5±9.2	29.0±8.3	0.097
Duration of diagnosis		11.2±3.0	11.6±1.8	0.290
BASFI		6.07±2.00	3.38±0.37	<0.001
BASDAI		6.28±1.34	5.42±1.16	<0.001
mSASSS		36.4±20.7	7.71±1.86	<0.001

There is no difference between severe AS patients and normal patients in age and sex distribution; however, the BASFI, BASDAI and mSASSS are higher in severe AS patients.

### 4: SNPs selection

**Figure 1:**
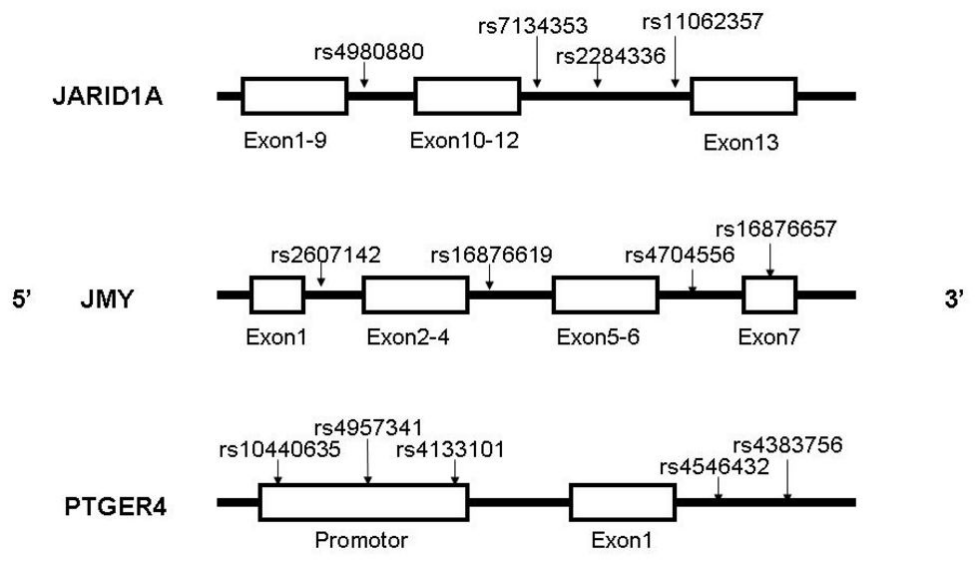
Positions of each selected tagSNP on the genes. The SNP rs16876657 is in the exon 7 of *JMY*, the SNP rs10440635, rs4957341, rs4133101 are in the promoter of *PTGER4*, other SNPs are all in introns.

The SNPs in this study included four in *JARID1A*, four in *JMY* and five in *PTGER4*. *JARID1A* is on chromosome 12, and *JMY* and *PTGER4* are both on chromosome 5. These SNPs are selected to serve as multi-marker tagging algorithm with criteria of r^2^ more than 0.8 and for all SNPs with minor allele frequency more than 20%; population is set as CHB (Chinese Han Beijing). We use the data download from hapmap to select the tagSNPs randomly. Haploview 4.2 software (Broad Institute, Cambridge, Massachusetts, USA) is used to in this procedure. Figure 1 shows the positions of each tagSNP.

### 5: DNA extraction and genotyping analysis

We use AxyPrep Blood Genomic DNA Miniprep kit (Axygen Biosciences, Union City, CA, USA) to isolate DNA from 2ml whole blood samples. We employ the chip-based matrix-assisted laser desorption ionization time-of-flight (MALDI-TOF) mass spectrometry technology to detect SNPs [26]. MassARRAY system is used in this procedure (Sequenom, San Diego, CA, USA). All SNPs in the control groups are successfully genotyped; rs7134353 and rs2284336 in *JARID1A* and rs10440635 in *PTGER4* in AS groups are 100% genotyped. The other SNPs are genotyped in 394 of 396 patients.

### 6: Statistical analysis

The Hardy-Weinberg equilibrium is tested for all 13 tagSNPs. We use chi-squared test and independent-samples t-test to compare the differences in age and gender between cases and controls. Comparisons of the distributions of the genotype, allele and haplotype frequencies are carried out using the chi-squared test. The relative risks are estimated as an odds ratio (OR) with a 95% confidence interval (CI). The p-values, OR, and 95% CI indicate in the text are used to estimate the significance of the contribution of corresponding genotype to disease risk. The p-values of genotypes indicate in the result tables are used to estimate the significance of the distribution of genotype between cases and controls. Different subgroups in cases are compared to controls separately. Bonferroni correction is needed. Due to the number of SNPs selected in each gene, p-value less than 0.01 is considered statistically significant after Bonferroni correction. The last genotype of each SNP is the major genotype and the last allele is the major allele. They are the reference groups. All three genotypes of each SNP are compared, p-value for individual genotypes are shown only if significant at 0.05 level (Table 3, details are shown in Table S1, Table S2 and Table S3). We compared the severe AS group to the entirety of the control group and then normal AS group to the entirety control group. Pearson’s chi-squared test is used to compare the constructed haplotypes. The SNPs which show significant differences between AS patients and controls are considered to be related to susceptibility to AS. The SNPs which p-value are less than 0.01 both in severe AS groups and normal AS groups are considered related to severity of AS. Statistical analyses are carried out with SPSS v.17.0 software package (IBM, Armonk, New York, USA). All three genotypes of each SNP are compared, and p-values are shown in the first line of each SNP. Additionally we compared the first two genotypes with the third one, showing the p-values if only they are significant (p<0.05) (Tables 3, S1, S2 and S3). We compare the severe AS group to the entirety of the control group and then normal AS group to the entirety of the control group.

**Table 3:** Positive SNPs in *JARID1A JMY* and *PTGER4* which are related to susceptibility to AS or severity of AS comparing all AS patients, severe AS patients and normal AS patients to the controls.

	SNP		All AS subjects cases / controls			Severe AS subjects cases / controls			Normal AS subjects cases / controls		
			frequencies	OR(95% CI)**^c^**	p	frequencies	OR(95% CI)	p	frequencies	OR(95% CI)	p
*JARID1A*	**rs2284336**	All^a^			0.001*			1.153E-6*			0.028#
	Genotype	TT	48/44	0.805(0.503~1.294)		4/44	0.243(0.083~0.718)		44/44	1.009(0.617~1.652)	
		CT	180/236	0.559(0.413~0.757)	1.858E-4*	28/236	0.302(0.180~0.505)	1.217E-6*	152/236	0.663(0.478~0.918)	0.018#
		CC	168/124	1**^b^**		50/124	1		118/124	1	
	Allele	T	276/324	0.799(0.652~0.979)	0.030#	36/324	0.420(0.283~0.624)	1.144E-5*	240/324	0.924(0.746~1.144)	0.469
		C	516/484	1		128/484	1		388/484	1	
	**rs11062357**	All			0.717			2.147E-8*			0.009*
	Genotype	CC	20/18	1.160(0.599~2.248)		18/18	7.546(3.584~15.890)	1.888E-9*	2/18	0.138(0.032~0.603)	0.002*
		CT	116/110	1.097(0.797~1.511)		28/110	1.780(1.027~3.087)		88/110	0.999(0.712~1.403)	
		TT	258/274	1		36/274	1		222/274	1	
	Allele	C	156/146	1.112(0.866~1.429)	0.405	64/146	2.884(2.010~4.140)	3.456E-9*	92/146	0.779(0.586~1.036)	0.086
		T	632/658	1		100/658	1		532/658	1	
*JMY*	**rs2607142**	All			0.064			3.825E-4*			0.007*
	genotype	AA	80/58	1.478(0.961~2.273)		8/58	0.417(0.182~0.956)	0.018#	72/58	2.079(1.315~3.289)	0.003*
		AG	200/230	0.892(0.647~1.229)		32/230	0.363(0.216~0.612)	1.809E-4*	168/230	1.187(0.832~1.696)	
		GG	114/116	1		42/116	1		72/116	1	
	allele	A	360/346	1.123(0.922~1.369)	0.250	48/346	0.553(0.384~0.795)	0.001*	312/346	1.335(1.082~1.647)	0.007*
		G	428/462	1		116/462	1		312/462	1	
	**rs16876619**	All			0.005*			0.017#			0.002*
	genotype	TT	46/22	2.257(1.297~3.925)	0.005*	6/22	1.120(0.427~2.941)		40/22	2.654(1.497~4.704)	0.001*
		CT	170/197	0.914(0.681~1.227)		26/197	0.490(0.291~0.825)	0.005*	144/197	1.066(0.778~1.461)	
		CC	178/183	1		50/183	1		128/183	1	
	allele	T	262/241	1.164(0.942~1.438)	0.160	38/241	0.232(0.161~0.335)	1.172E-16*	224/241	1.308(1.047~1.634)	0.018#
		C	526/563	1		382/563	1		400/563	1	
	**rs16876657**	All			**0.012#**			0.006*			0.051
	genotype	GG	2/0	N/A		0/0	N/A		2/0	N/A	
		AG	52/81	0.609(0.416~0.891)	0.009*	6/81	0.311(0.130~0.742)	0.006*	46/81	0.692(0.465~1.030)	
		AA	340/321	1		76/321	1		264/321	1	
	allele	G	56/81	0.683(0.478~0.975)	0.035#	6/81	0.339(0.145~0.791)	0.009*	50/81	0.778(0.538~1.125)	0.181
		A	732/723	1		158/723	1		574/723	1	
*PTGER4*	**rs10440635**	All			0.523			8.649E-6*			9.282E-5*
		AA	20/28	0.677(0.369~1.244)		18/28	4.899(2.389~10.044)	1.126E-6*	2/28	0.079(0.018~0.335)	1.763E-5*
		AG	148/148	0.973(0.723~1.310)		36/150	1.881(1.097~3.227)	0.014#	112/150	0.857(0.626~1.174)	
		GG	228/226	1		28/226	1		200/226	1	
		A	188/204	0.915(0.729~1.150)	0.448	72/204	2.302(1.627~3.256)	1.667E-6*	116/204	0.666(0.516~0.861)	0.002*
		G	604/600	1		92/600	1		512/600	1	

a: ”All” means the p value that we compare all the three genotypes using 3×2 chi squared method.

b: The last lines of genotypes or alleles are the major genotypes or the major alleles. The other genotypes or alleles are compared to them. p-value for individual genotypes are shown only if significant at 0.05 level. The relative risk associated with major genotypes and major alleles is estimated as an odds ratio (OR) with a 95% confidence interval (CI).

c: OR (95% CI) are adjusted by age and sex using binary logistic regression analysis.

# indicates p-value is less than 0.05 but cannot pass Bonferroni correction which shows marginal significant difference.

* indicates p-value is less than 0.01 which shows significant difference after Bonferroni correction.

The details of all the 13 SNPs are summarized in Table S1 (*JARID1A*), Table S2 (*JMY*) and Table S3 (*PTGER4*)

### 7: Ethics statement

The blood samples of both AS patients and controls used in this study are part of samples taken for diagnostic tests. During the collection and use of DNA samples, clinical data guidelines, regulations of the local Ethics Committee and the Helsinki Declaration in 1975 are followed. Written informed consents were obtained from all the patients and subjects (or their parents in the case of two patients less than 18 years old). The study procedure is approved by our Institutional Review Board. The full name of the IRB is ethics committee of Chinese PLA general hospital.

## Results

### 1: Clinical features

Among the 396 AS patients, the mean BASFI is 3.94±1.45 (mean±standard deviation). The mean BASDAI is 5.60±1.25. The mean mSASSS is 13.7±15.0. When comparing the severe AS to the normal AS patient groups, there is no significant difference in sex (p=0.220), age (p=0.097), and duration since diagnosis (p= 0.290) (Table 2). The BASFI is higher in severe AS group (6.07±2.00) than normal AS group (3.38±0.37) (p-value<0.001), reflecting poorer function of patients in the severe AS group. The BASDAI is similarly higher in the severe AS group (6.28±1.34) than normal AS group (5.42±1.16) (p-value<0.001), reflecting higher disease activity. The pattern holds for mSASSS (36.4±20.7 versus 7.71±1.86, p-value<0.001), signifying more radiographic changes in the severe AS patients.

### 2: Genotype and allele

The statistically significant SNPs of these three genes related to susceptibility to AS and severity of AS are summarized in Table 3. The details of genotype and allele distributions for *JARID1A, JMY* and *PTGER4* are summarized in Table S1, Table S2 and Table S3 respectively. The genotype frequencies of these 13 tagSNPs are in Hardy-Weinberg equilibrium case groups and control groups. SNPs in *JARID1A* are compared between all AS patients, severe AS patients, and normal AS patients versus the control subjects (Table S1). The rs7134353 SNP shows significant difference when comparing severe AS patients to controls, with AA genotype higher in severe AS than in controls (p=2.241×10^-4^). The rs2284336 SNP shows significant difference when comparing all AS patients to controls, with CT genotype lower in all AS than in controls (p=1.858×10^-4^); this SNP also shows significant difference when comparing severe AS patients to controls, with CT genotype lower in severe AS patients than in controls (p=1.217×10^-6^), and T allele lower in severe AS than in controls (p=1.144×10^-5^). The rs11062357 SNP shows significant difference when comparing severe AS patients to controls, with CC genotype higher in severe AS than in controls (p=1.888×10^-9^) and C allele higher in severe AS than in controls (p=3.456×10^-9^); this SNP also shows significant difference when comparing normal AS to controls, with CC genotype lower in normal AS than in controls (p=0.002).

The SNPs in *JMY* are compared between all AS patients, severe AS patients, and normal AS patients versus the control subjects (Table S2). The rs2607142 SNP shows significant difference when comparing severe AS patients to controls, with AG genotype lower in severe AS than in controls (p=1.809×10^-4^) and A allele lower in severe AS than in controls (p=0.001); This SNP also show significant difference when comparing normal AS to controls, with AA genotype higher in normal AS than in controls (p=0.003) and A allele higher in normal AS than in controls (p=0.007). The rs16876619 SNP shows significant difference when comparing all AS patients to controls, with TT genotype higher in all AS than in controls (p=0.005); this SNP also shows significant difference when comparing severe AS patients to controls, with CT genotype lower in severe AS than in controls (p=0.005), T allele lower in severe AS than in controls(p=1.172×10^-16^) ; and this SNP shows significant difference when comparing normal AS patients to controls, with TT genotype higher in normal AS than in controls (p=0.001). Additionally CT genotype is lower than TT genotype (p=0.001). The rs4704556 SNP shows significant difference when comparing severe AS patients to controls, with CC genotype higher in severe AS than in controls (p=5.844×10^-7^), C allele is higher in severe AS than in controls (p=2.249×10^-7^). The rs16876657 SNP shows significant difference when comparing all AS patients to controls, with AG genotype lower in all AS than in controls (p=0.009); this SNP also shows significant difference when comparing severe AS patients to controls, with AG genotype lower in severe AS than in controls (p=0.006), G allele lower in severe AS than in controls (p=0.009)

The SNPs in *PTGER4* are compared between all AS patients, severe AS patients, and normal AS patients versus the control subjects (Table S3). The rs10440635 SNP shows significant difference when comparing severe AS patients to controls, with AA genotype higher in severe AS than in controls (p=1.126×10^-6^), A allele higher in severe AS than in controls (p=1.667×10^-6^); this SNP also shows significant difference when comparing normal AS to controls, with AA genotype lower in normal AS than in controls (p=1.763×10^-5^), A allele lower in normal AS than in controls (p=0.002). The rs4957341 SNP shows significant difference when comparing severe AS to controls, with AA genotype higher in severe AS than in controls (p=0.003).

Referring to the data from Hapmap: in *JARID1A*, rs2284336 is in 100% LD with rs11062385; in *JMY*, rs16876657 is related to susceptibility of AS. These results can support the former researches in western descendent. *PTGER4* shows no association with susceptibility of AS in Han Chinese. The rs11062357 SNP in *JARID1A*, the rs2607142 SNP in *JMY* and rs10440635 in *PTGER4* are related to severity of AS.

### 3: Haplotype

**Figure 2:**
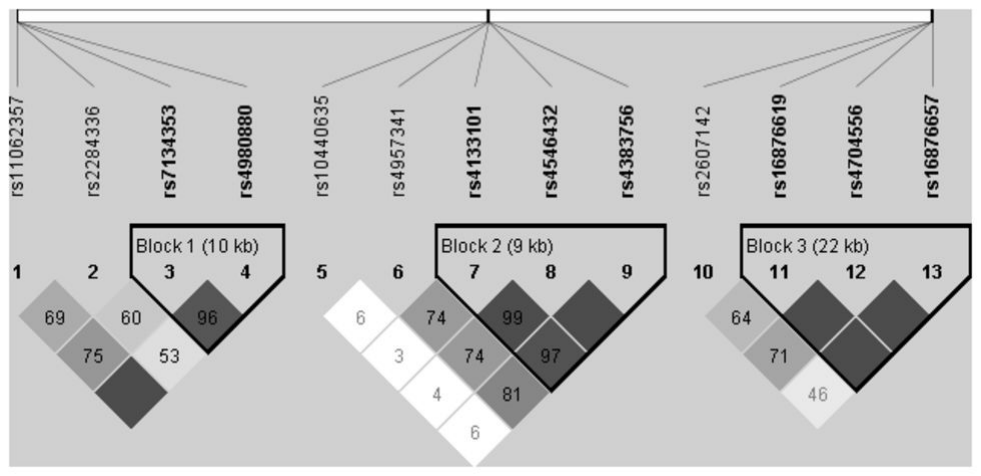
Linkage disequilibrium (LD) map comparing all AS patients and controls. Darker color indicates higher linkage disequilibrium (LD), lighter color indicates less LD. Numbers in the squares indicate correlation coefficient (R^2^) value. The left part of the picture contains 4 SNPs (from rs11062357 to rs4980880). They are from *JARID1A*. The middle part of the picture contains 5 SNPs (from rs10440635 to rs4383756). They are from *PTGER4*. The right part of the picture contains 4 SNPs (from rs2607142 to rs16876657). They are from *JMY*. Haplotypes are constructed from the darker blocks (high linkage disequilibrium). Haplotypes are constructed from each blocks, the details of haplotypes are summarized in Table S4. Block 2 contains rs4133101 rs4546432 and rs4383756 SNPs in *PTGER4*. CTT frequency is higher than controls (p=6.266×10^-8^).

Linkage disequilibrium (LD) maps of the 13 tagSNPs of *JARID1A, JMY* and *PTGER4* comparing all AS patients, severe AS patients, and normal AS patients to controls subjects are shown in Figure 2, Figure S1 and Figure S2, respectively. These figures have only a little difference, only Figure 2 is shown in the text comparing all AS patients to controls. Figure S1 and Figure S2 are shown in the appendices comparing severe patients and normal patients to controls separately. Analyses of constructed haplotypes are shown in Table S4, Table S5 and Table S6 in appendices. When comparing all AS to controls (Table S4), the rs4133101,rs4546432 and rs4383756 SNPs in *PTGER4* show significant difference, the haplotype CTT frequency is higher than controls (p=6.266×10^-8^). When comparing severe AS to controls (Table S5), the rs7134353 and rs4980880 SNPs in *JARID1A* show significant difference, the haplotype TT is lower than controls (p=4.136×10^-4^). The rs16876619 and rs4704556 SNPs in *JMY* show significant difference, the haplotype CC is higher than controls (p=2.682×10-7); the haplotype CT is lower than controls (p=4.660×10^-5^). When comparing normal AS to controls (Table S6), the rs16876619, rs4704556 and rs16876657 SNPs in *JMY* shows marginal significant difference, the haplotype TTA is marginal significant higher than controls but cannot pass Bonferroni correction (p=0.028).

In conclusion, *PTGER4* is related to susceptibility to AS; *JARID1A* and *JMY* are related to severity of AS.

## Discussion

Three genes studied include *JARID1A, JMY*, and *PTGER4*. *JARID1A* regulates gene expression and is involved in tumorigenesis; it has been best studied in association with breast cancer [8,10,11]. *JMY* encodes a transcription co-factor that augments the p53 tumor suppressor response [12]. *PTGER4* encodes a prostaglandin receptor, and its down-regulation halts certain cell proliferation [13,14]. *JARID1A, JMY*, and *PTGER4* have been linked to AS in GWAS in patients of western European descents [15-17]; we focus on particular SNPs of these genes in the Chinese Han population.

In comparing 396 AS patients and 404 healthy controls, we find that Frequencies of different genotypes and alleles are analyzed among the different severity AS patients and the controls. The rs2284336 SNP in *JARID1A*, the rs16876619 and rs16876657 SNPs in *JMY* are associated with susceptibility of AS. The rs11062357 SNP in *JARID1A*, the rs2607142 SNP in *JMY* and rs10440635 in *PTGER4* are related to severity of AS. Haplotype analyses indicate *PTGER4* is related to susceptibility to AS; *JARID1A* and *JMY* are related to severity of AS.

*JARID1A* is recently found to interact physically and functionally with the Polycomb complex. This protein can influence the differentiation of CD4+ T-cells [27]. Other research supports that *JARID1A* plays an important role in regulation of immune cells such as CD56+ NK cells, CD8+ T cells, dendritic cells and CD34+ cells [28]. In addition to susceptibility to AS, the rs2284336 SNP in *JARID1A* may have association with other autoimmune diseases.

With its influence on p53, *JMY* can affects apoptosis during the DNA damage response [29]. *PTGER4*-encoded EP4R signaling mediates ultraviolet induced immunosuppression through modulation of regulatory T cells and RANKL expression [30]; furthermore, EP4R can restrict the survival of immature B cells [31]. The exact mechanisms how JMY and *PTGER4-*encoded EP4R’s effects on the immune system can influence the AS disease processes remain to be elucidated.

Histone demethylase JARID1A is found to be related to susceptibility to AS in the western descendent [17]. This mechanism should be investigated in Chinese Han population. The severe AS patient’s subgroup has 82 patients. This may be a low power data. However, these patients are in the nature course of the disease with only non-steroidal anti-inflammatory drugs treatments and have severe deformity, which may be impossible to be found in the western countries due to their regular treatments. We are the first to divide the AS patients into subgroups due to severity. Severity is related to prognosis of AS which is important for patients and therapeutic method choice.

In conclusion, in Chinese patients, *JARID1A*, *JMY* and *PTGER4* are related to susceptibility to AS; *JARID1A* and *JMY* are related to severity of AS. These findings may lead to full understanding of the genetic and molecular pathogenesis of AS. Of clinical relevance, the specific SNPs in these genes can be used to guide genetic analysis and counseling, medical and surgical treatment options, and ultimate prognosis. Further studies are needed to elucidate the molecular roles these genes play in AS.

## Supporting Information

Figure S1 **Linkage disequilibrium map comparing severe AS patients and controls.** The distribution and position of SNPs are the same as Figure 2. Haplotypes are constructed from the darker blocks (high linkage disequilibrium). Haplotypes are constructed from each blocks, the details of haplotypes are summarized in Table S5. Block 1 contains rs7134353 and rs4980880 SNPs in JARID1A. TT is lower than controls (p=4.136×10^-4^). Block 3 contains rs16876619 and rs4704556 SNPs in *JMY*. CC is higher than controls (p=2.682×10^-7^). CT is lower than controls (p=4.660×10^-5^).
(TIF)

Figure S2 **Linkage disequilibrium map comparing normal AS patients and controls.** The distribution and position of SNPs are the same as Figure 2. Haplotypes are constructed from each blocks, the details of haplotypes are summarized in Table S6. Block 3 contains rs16876619, rs4704556 and rs16876657 SNPs in *JMY*. TTA is marginal significant higher than controls but cannot pass Bonferroni correction.
(TIF)

Table S1 **Genotype and allele frequencies of *JARID1A* SNPs among all AS patients, severe AS patients, normal AS patients versus controls.** SNPs in *JARID1A* are compared between all AS patients, severe AS patients, and normal AS patients versus the control subjects. P-value for each SNP is shown, and p-value for individual genotypes are shown only if significant at 0.05 level. # indicates P-value is less than 0.05 but cannot pass Bonferroni correction which shows marginal significant difference. *indicates P-value is less than 0.01 which shows significant difference after Bonferroni correction. The rs7134353 SNP shows significant difference when comparing severe AS patients to controls, AA genotype is higher than controls (p=2.241×10^-4^). The rs2284336 SNP shows significant difference when comparing all AS patients to controls, the CT genotype is lower than controls (p=1.858×10^-4^); this SNP also shows significant difference when comparing severe AS patients to controls, CT genotype is lower than controls (p=1.217×10^-6^), and T allele is lower than controls (p=1.144×10^-5^). The rs11062357 SNP shows significant difference when comparing severe AS patients to controls, CC genotype is higher than controls (p=1.888×10^-9^) and C allele is higher than controls (p=3.456×10^-9^); this SNP also shows significant difference when comparing normal AS to controls, CC genotype is lower than controls (p=0.002).
(DOCX)

Table S2 **Genotype and allele frequencies of *JMY* SNPs among all AS patients, severe AS patients, normal AS patients versus controls.** SNPs in *JMY* are compared between all AS patients, severe AS patients, and normal AS patients versus the control subjects. The rs2607142 SNP shows significant difference when comparing severe AS patients to controls, AG genotype is lower than controls (p=1.809×10^-4^) and A allele is lower than controls (p=0.001); This SNP also show significant difference when comparing normal AS to controls, AA genotype is higher than controls (p=0.003) and A allele is higher than controls (p=0.007). The rs16876619 SNP shows significant difference when comparing all AS patients to controls, TT genotype is higher than controls (p=0.005); this SNP also shows significant difference when comparing severe AS patients to controls, CT genotype is lower than controls (p=0.005), T allele is lower than controls(p=1.172×10^-16^) ; And this SNP shows significant difference when comparing normal AS patients to controls, TT genotype is higher than controls (p=0.001). Additionally CT genotype is lower than TT genotype (p=0.001). The rs4704556 SNP shows significant difference when comparing severe AS patients to controls, CC genotype is higher than controls (p=5.844×10^-7^), C allele is higher than controls (p=2.249×10^-7^). The rs16876657 SNP shows significant difference when comparing all AS patients to controls, AG genotype is lower than controls (p=0.009); this SNP also shows significant difference when comparing severe AS patients to controls AG genotype is lower than controls (p=0.006), G allele is lower than controls (p=0.009).
(DOCX)

Table S3 **Genotype and allele frequencies of *PTGER4* SNPs among all AS patients, severe AS patients, normal AS patients versus controls.** SNPs in *PTGER4* are compared between all AS patients, severe AS patients, and normal AS patients versus the control subjects. The rs10440635 SNP shows significant difference when comparing severe AS patients to controls, AA genotype is higher than controls (p=1.126×10^-6^), A allele is higher than controls (p=1.667×10^-6^); this SNP also shows significant difference when comparing normal AS to controls, AA genotype is lower than controls (p=1.763×10^-5^), A allele is lower than controls (p=0.002). The rs4957341 SNP shows significant difference when comparing severe AS to controls, AA genotype is higher than controls (p=0.003).
(DOCX)

Table S4 **Haplotype analysis comparing all AS patients to controls.** Haplotypes are constructed due to LD map (Figure 2). Case ratio means in the case group, the frequency of this kind of haplotype vs. other kinds of haplotype; control ratio means in the control group, the frequency of this kind of haplotype vs. other kinds of haplotype. Block 2 contains rs4133101 rs4546432 and rs4383756 SNPs in PTGER4. CTT frequency is higher than controls (p=6.266×10-8).
(DOCX)

Table S5 **Haplotype analysis comparing severe AS patients to controls.** Haplotypes are constructed due to Figure A1. Case ratio means in the severe AS group, the frequency of this kind of haplotype vs. other kinds of haplotype; control ratio means in the control group, the frequency of this kind of haplotype vs. other kinds of haplotype. Block 1 contains rs7134353 and rs4980880 SNPs in JARID1A. TT is lower than controls (p=4.136×10-4). Block 3 contains rs16876619 and rs4704556 SNPs in JMY. CC is higher than controls (p=2.682×10-7). CT is lower than controls (p=4.660×10-5).
(DOCX)

Table S6 **Haplotype analysis comparing severe AS patients to controls.** Haplotypes are constructed. Case ratio means in the case group, the frequency of this kind of haplotype vs. other kinds of haplotype; control ratio means in the control group, the frequency of this kind of haplotype vs. other kinds of haplotype. Block 3 contains rs16876619, rs4704556 and rs16876657 SNPs in JMY. TTA is marginal significant higher than controls but cannot pass Bonferroni correction.
(DOCX)
