# Supplementary figures and images for: JARID1A, JMY, and PTGER4 Polymorphisms Are Related to Ankylosing Spondylitis in Chinese Han Patients: A Case-Control Study
Source: PLoS One. 2013 Sep 19;8(9):e74794. doi: 10.1371/journal.pone.0074794 (PMC3777963; doi:10.1371/journal.pone.0074794)

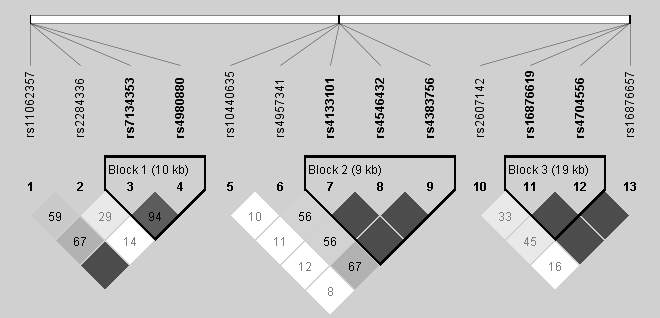

Supplement: Figure S1 — Linkage disequilibrium map comparing severe AS patients and controls. The distribution and position of SNPs are the same as Figure 2. Haplotypes are constructed from the darker blocks (high linkage disequilibrium). Haplotypes are constructed from each blocks, the details of haplotypes are summarized in Table S5. Block 1 contains rs7134353 and rs4980880 SNPs in JARID1A. TT is lower than controls (p=4.136×10^-4^). Block 3 contains rs16876619 and rs4704556 SNPs in JMY. CC is higher than controls (p=2.682×10^-7^). CT is lower than controls (p=4.660×10^-5^). (TIF) [file pone.0074794.s001.tif]

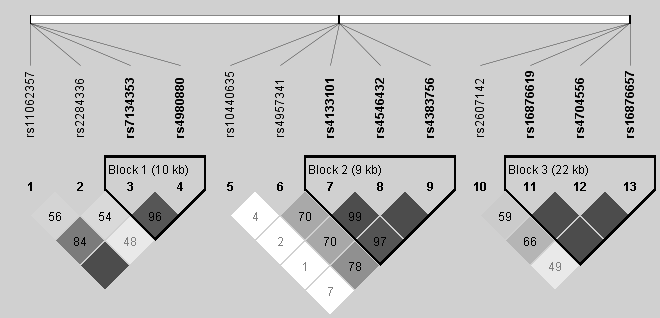

Supplement: Figure S2 — Linkage disequilibrium map comparing normal AS patients and controls. The distribution and position of SNPs are the same as Figure 2. Haplotypes are constructed from each blocks, the details of haplotypes are summarized in Table S6. Block 3 contains rs16876619, rs4704556 and rs16876657 SNPs in JMY. TTA is marginal significant higher than controls but cannot pass Bonferroni correction. (TIF) [file pone.0074794.s002.tif]
