# Supplementary material for: JARID1A, JMY, and PTGER4 Polymorphisms Are Related to Ankylosing Spondylitis in Chinese Han Patients: A Case-Control Study
Source: PLoS One. 2013 Sep 19;8(9):e74794. doi: 10.1371/journal.pone.0074794 (PMC3777963; doi:10.1371/journal.pone.0074794)
Supplement: Table S1 — Genotype and allele frequencies of JARID1A SNPs among all AS patients, severe AS patients, normal AS patients versus controls. SNPs in JARID1A are compared between all AS patients, severe AS patients, and normal AS patients versus the control subjects. P-value for each SNP is shown, and p-value for individual genotypes are shown only if significant at 0.05 level. # indicates P-value is less than 0.05 but cannot pass Bonferroni correction which shows marginal significant difference. *indicates P-value is less than 0.01 which shows significant difference after Bonferroni correction. The rs7134353 SNP shows significant difference when comparing severe AS patients to controls, AA genotype is higher than controls (p=2.241×10^-4^). The rs2284336 SNP shows significant difference when comparing all AS patients to controls, the CT genotype is lower than controls (p=1.858×10^-4^); this SNP also shows significant difference when comparing severe AS patients to controls, CT genotype is lower than controls (p=1.217×10^-6^), and T allele is lower than controls (p=1.144×10^-5^). The rs11062357 SNP shows significant difference when comparing severe AS patients to controls, CC genotype is higher than controls (p=1.888×10^-9^) and C allele is higher than controls (p=3.456×10^-9^); this SNP also shows significant difference when comparing normal AS to controls, CC genotype is lower than controls (p=0.002). (DOCX) [file pone.0074794.s003.docx]

Table S1. Genotype and allele frequencies of *JARID1A* SNPs among all AS patients, severe AS patients, normal AS patients versus controls.

| SNP |  | All AS subjects cases / controls | |  | Severe AS subjects cases / controls | |  | Normal AS subjects cases / controls | |  |
| --- | --- | --- | --- | --- | --- | --- | --- | --- | --- | --- |
|  |  | frequencies | OR(95% CI) | p | frequencies | OR(95% CI) | p | frequencies | OR(95% CI) | p |
| **rs4980880** | All |  |  | 0.148 |  |  | 0.727 |  |  | 0.094 |
| Genotype | GG | 4/10 | 0.415(0.128~1.339) |  | 2/10 | 1.204(0.255~5.685) |  | 2/10 | 0.250(0.054~1.154) |  |
|  | GT | 76/64 | 1.249(0.865~1.804) |  | 16/64 | 1.321(0.714~2.444) |  | 60/64 | 1.227(0.831~1.812) |  |
|  | TT | 314/328 | 1 |  | 64/328 | 1 |  | 250/328 | 1 |  |
| Allele | G | 84/84 | 1.023(0.743~1.408) | 0.890 | 20/84 | 0.822(0.454~1.487) | 0.516 | 64/84 | 1.238(0.894~1.714) | 0.199 |
|  | T | 704/720 | 1 |  | 144/720 | 1 |  | 560/720 |  |  |
|  |  |  |  |  |  |  |  |  |  |  |
| **rs7134353** | All |  |  | 0.032# |  |  | **0.001*** |  |  | 0.092 |
| Genotype | AA | 102/74 | 1.388(0.938~2.053) |  | 30/74 | 3.339(1.674~6.661) | **2.241E-4*** | 72/74 | 1.136(0.751~1.719) |  |
|  | AT | 170/200 | 0.874(0.633~1.205) |  | 37/200 | 1.592(0.837~3.031) |  | 133/200 | 0.788(0.562~1.106) |  |
|  | TT | 124/128 | 1 |  | 15/128 | 1 |  | 109/128 | 1 |  |
| Allele | A | 374/348 | 1.171(0.961~1.426) | 0.117 | 97/348 | 1.190(0.708~2.001) | 0.510 | 277/348 | 1.034(0.838~1.276) | 0.755 |
|  | T | 418/456 | 1 |  | 67/456 | 1 |  | 351/456 | 1 |  |
|  |  |  |  |  |  |  |  |  |  |  |
| **rs2284336** | All |  |  | **0.001*** |  |  | **1.153E-6*** |  |  | 0.028# |
| Genotype | TT | 48/44 | 0.805(0.503~1.294) |  | 4/44 | 0.243(0.083~0.718) |  | 44/44 | 1.009(0.617~1.652) |  |
|  | CT | 180/236 | 0.559(0.413~0.757) | **1.858E-4*** | 28/236 | 0.302(0.180~0.505) | **1.217E-6*** | 152/236 | 0.663(0.478~0.918) | 0.018# |
|  | CC | 168/124 | 1 |  | 50/124 | 1 |  | 118/124 | 1 |  |
| Allele | T | 276/324 | 0.799(0.652~0.979) | 0.030# | 36/324 | 0.420(0.283~0.624) | **1.144E-5*** | 240/324 | 0.924(0.746~1.144) | 0.469 |
|  | C | 516/484 | 1 |  | 128/484 | 1 |  | 388/484 | 1 |  |
|  |  |  |  |  |  |  |  |  |  |  |
| **rs11062357** | All |  |  | 0.717 |  |  | **2.147E-8*** |  |  | **0.009*** |
| Genotype | CC | 20/18 | 1.160(0.599~2.248) |  | 18/18 | 7.546(3.584~15.890) | **1.888E-9*** | 2/18 | 0.138(0.032~0.603) | **0.002*** |
|  | CT | 116/110 | 1.097(0.797~1.511) |  | 28/110 | 1.780(1.027~3.087) |  | 88/110 | 0.999(0.712~1.403) |  |
|  | TT | 258/274 | 1 |  | 36/274 | 1 |  | 222/274 | 1 |  |
| Allele | C | 156/146 | 1.112(0.866~1.429) | 0.405 | 64/146 | 2.884(2.010~4.140) | **3.456E-9*** | 92/146 | 0.779(0.586~1.036) | 0.086 |
|  | T | 632/658 | 1 |  | 100/658 | 1 |  | 532/658 | 1 |  |

SNPs in *JARID1A* are compared between all AS patients, severe AS patients, and normal AS patients versus the control subjects. P-value for each SNP is shown, and p-value for individual genotypes are shown only if significant at 0.05 level. # indicates P-value is less than 0.05 but cannot pass Bonferroni correction which shows marginal significant difference. *indicates P-value is less than 0.01 which shows significant difference after Bonferroni correction. The rs7134353 SNP shows significant difference when comparing severe AS patients to controls, AA genotype is higher than controls (p=2.241×10^-4^). The rs2284336 SNP shows significant difference when comparing all AS patients to controls, the CT genotype is lower than controls (p=1.858×10^-4^); this SNP also shows significant difference when comparing severe AS patients to controls, CT genotype is lower than controls (p=1.217×10^-6^), and T allele is lower than controls (p=1.144×10^-5^). The rs11062357 SNP shows significant difference when comparing severe AS patients to controls, CC genotype is higher than controls (p=1.888×10^-9^) and C allele is higher than controls (p=3.456×10^-9^); this SNP also shows significant difference when comparing normal AS to controls, CC genotype is lower than controls (p=0.002).
