# Supplementary material for: JARID1A, JMY, and PTGER4 Polymorphisms Are Related to Ankylosing Spondylitis in Chinese Han Patients: A Case-Control Study
Source: PLoS One. 2013 Sep 19;8(9):e74794. doi: 10.1371/journal.pone.0074794 (PMC3777963; doi:10.1371/journal.pone.0074794)
Supplement: Table S2 — Genotype and allele frequencies of JMY SNPs among all AS patients, severe AS patients, normal AS patients versus controls. SNPs in JMY are compared between all AS patients, severe AS patients, and normal AS patients versus the control subjects. The rs2607142 SNP shows significant difference when comparing severe AS patients to controls, AG genotype is lower than controls (p=1.809×10^-4^) and A allele is lower than controls (p=0.001); This SNP also show significant difference when comparing normal AS to controls, AA genotype is higher than controls (p=0.003) and A allele is higher than controls (p=0.007). The rs16876619 SNP shows significant difference when comparing all AS patients to controls, TT genotype is higher than controls (p=0.005); this SNP also shows significant difference when comparing severe AS patients to controls, CT genotype is lower than controls (p=0.005), T allele is lower than controls(p=1.172×10^-16^) ; And this SNP shows significant difference when comparing normal AS patients to controls, TT genotype is higher than controls (p=0.001). Additionally CT genotype is lower than TT genotype (p=0.001). The rs4704556 SNP shows significant difference when comparing severe AS patients to controls, CC genotype is higher than controls (p=5.844×10^-7^), C allele is higher than controls (p=2.249×10^-7^). The rs16876657 SNP shows significant difference when comparing all AS patients to controls, AG genotype is lower than controls (p=0.009); this SNP also shows significant difference when comparing severe AS patients to controls AG genotype is lower than controls (p=0.006), G allele is lower than controls (p=0.009). (DOCX) [file pone.0074794.s004.docx]

Table S2. Genotype and allele frequencies of *JMY* SNPs among all AS patients, severe AS patients, normal AS patients versus controls.

| SNP |  | All AS subjects cases / controls | |  | Severe AS subjects cases / controls | |  | Normal AS subjects cases / controls | |  |
| --- | --- | --- | --- | --- | --- | --- | --- | --- | --- | --- |
|  |  | frequencies | OR(95% CI) | p | frequencies | OR(95% CI) | p | frequencies | OR(95% CI) | p |
| **rs2607142** | All |  |  | 0.064 |  |  | **3.825E-4*** |  |  | **0.007*** |
| genotype | AA | 80/58 | 1.478(0.961~2.273) |  | 8/58 | 0.417(0.182~0.956) | **0.018#** | 72/58 | 2.079(1.315~3.289) | **0.003*** |
|  | AG | 200/230 | 0.892(0.647~1.229) |  | 32/230 | 0.363(0.216~0.612) | **1.809E-4*** | 168/230 | 1.187(0.832~1.696) |  |
|  | GG | 114/116 | 1 |  | 42/116 | 1 |  | 72/116 | 1 |  |
| allele | A | 360/346 | 1.123(0.922~1.369) | 0.250 | 48/346 | 0.553(0.384~0.795) | **0.001*** | 312/346 | 1.335(1.082~1.647) | **0.007*** |
|  | G | 428/462 | 1 |  | 116/462 | 1 |  | 312/462 | 1 |  |
|  |  |  |  |  |  |  |  |  |  |  |
| **rs16876619** | All |  |  | **0.005*** |  |  | 0.017# |  |  | **0.002*** |
| genotype | TT | 46/22 | 2.257(1.297~3.925) | **0.005*** | 6/22 | 1.120(0.427~2.941) |  | 40/22 | 2.654(1.497~4.704) | **0.001*** |
|  | CT | 170/197 | 0.914(0.681~1.227) |  | 26/197 | 0.490(0.291~0.825) | **0.005*** | 144/197 | 1.066(0.778~1.461) |  |
|  | CC | 178/183 | 1 |  | 50/183 | 1 |  | 128/183 | 1 |  |
| allele | T | 262/241 | 1.164(0.942~1.438) | 0.160 | 38/241 | 0.232(0.161~0.335) | **1.172E-16*** | 224/241 | 1.308(1.047~1.634) | 0.018# |
|  | C | 526/563 | 1 |  | 382/563 | 1 |  | 400/563 | 1 |  |
|  |  |  |  |  |  |  |  |  |  |  |
| **rs4704556** | All |  |  | 0.470 |  |  | **3.427E-8*** |  |  | 0.404 |
| genotype | CC | 82/70 | 1.182(0.777~1.801) |  | 38/70 | 5.817(2.706~12.503) | **5.844E-7*** | 44/70 | 0.705(0.441~1.128) |  |
|  | CT | 206/221 | 0.968(0.697~1.343) |  | 34/221 | 1.624(0.771~3.423) |  | 172/221 | 0.897(0.605~1.252) |  |
|  | TT | 106/111 | 1 |  | 10/111 | 1 |  | 96/111 | 1 |  |
| allele | C | 370/361 | 1.086(0.892~1.323) | 0.411 | 110/361 | 2.500(1.754~3.562) | **2.249E-7*** | 260/361 | 0.877(0.710~1.083) | 0.221 |
|  | T | 418/443 | 1 |  | 54/443 | 1 |  | 364/443 | 1 |  |
|  |  |  |  |  |  |  |  |  |  |  |
| **rs16876657** | All |  |  | **0.012#** |  |  | **0.006*** |  |  | 0.051 |
| genotype | GG | 2/0 | N/A |  | 0/0 | N/A |  | 2/0 | N/A |  |
|  | AG | 52/81 | 0.609(0.416~0.891) | **0.009*** | 6/81 | 0.311(0.130~0.742) | **0.006*** | 46/81 | 0.692(0.465~1.030) |  |
|  | AA | 340/321 | 1 |  | 76/321 | 1 |  | 264/321 | 1 |  |
| allele | G | 56/81 | 0.683(0.478~0.975) | 0.035# | 6/81 | 0.339(0.145~0.791) | **0.009*** | 50/81 | 0.778(0.538~1.125) | 0.181 |
|  | A | 732/723 | 1 |  | 158/723 | 1 |  | 574/723 | 1 |  |

SNPs in *JMY* are compared between all AS patients, severe AS patients, and normal AS patients versus the control subjects. The rs2607142 SNP shows significant difference when comparing severe AS patients to controls, AG genotype is lower than controls (p=1.809×10^-4^) and A allele is lower than controls (p=0.001); This SNP also show significant difference when comparing normal AS to controls, AA genotype is higher than controls (p=0.003) and A allele is higher than controls (p=0.007). The rs16876619 SNP shows significant difference when comparing all AS patients to controls, TT genotype is higher than controls (p=0.005); this SNP also shows significant difference when comparing severe AS patients to controls, CT genotype is lower than controls (p=0.005), T allele is lower than controls(p=1.172×10^-16^) ; And this SNP shows significant difference when comparing normal AS patients to controls, TT genotype is higher than controls (p=0.001). Additionally CT genotype is lower than TT genotype (p=0.001). The rs4704556 SNP shows significant difference when comparing severe AS patients to controls, CC genotype is higher than controls (p=5.844×10^-7^), C allele is higher than controls (p=2.249×10^-7^). The rs16876657 SNP shows significant difference when comparing all AS patients to controls, AG genotype is lower than controls (p=0.009); this SNP also shows significant difference when comparing severe AS patients to controls AG genotype is lower than controls (p=0.006), G allele is lower than controls (p=0.009)
