# Supplementary material for: JARID1A, JMY, and PTGER4 Polymorphisms Are Related to Ankylosing Spondylitis in Chinese Han Patients: A Case-Control Study
Source: PLoS One. 2013 Sep 19;8(9):e74794. doi: 10.1371/journal.pone.0074794 (PMC3777963; doi:10.1371/journal.pone.0074794)
Supplement: Table S3 — Genotype and allele frequencies of PTGER4 SNPs among all AS patients, severe AS patients, normal AS patients versus controls. SNPs in PTGER4 are compared between all AS patients, severe AS patients, and normal AS patients versus the control subjects. The rs10440635 SNP shows significant difference when comparing severe AS patients to controls, AA genotype is higher than controls (p=1.126×10^-6^), A allele is higher than controls (p=1.667×10^-6^); this SNP also shows significant difference when comparing normal AS to controls, AA genotype is lower than controls (p=1.763×10^-5^), A allele is lower than controls (p=0.002). The rs4957341 SNP shows significant difference when comparing severe AS to controls, AA genotype is higher than controls (p=0.003). (DOCX) [file pone.0074794.s005.docx]

Table S3. Genotype and allele frequencies of *PTGER4* SNPs among all AS patients, severe AS patients, normal AS patients versus controls.

| SNP |  | All AS subjects cases / controls | |  | Severe AS subjects cases / controls | |  | Normal AS subjects cases / controls | |  |
| --- | --- | --- | --- | --- | --- | --- | --- | --- | --- | --- |
|  |  | frequencies | OR(95% CI) | p | frequencies | OR(95% CI) | p | frequencies | OR(95% CI) | p |
| **rs10440635** | All |  |  | 0.523 |  |  | **8.649E-6*** |  |  | **9.282E-5*** |
|  | AA | 20/28 | 0.677(0.369~1.244) |  | 18/28 | 4.899(2.389~10.044) | **1.126E-6*** | 2/28 | 0.079(0.018~0.335) | **1.763E-5*** |
|  | AG | 148/148 | 0.973(0.723~1.310) |  | 36/150 | 1.881(1.097~3.227) | 0.014# | 112/150 | 0.857(0.626~1.174) |  |
|  | GG | 228/226 | 1 |  | 28/226 | 1 |  | 200/226 | 1 |  |
|  | A | 188/204 | 0.915(0.729~1.150) | 0.448 | 72/204 | 2.302(1.627~3.256) | **1.667E-6*** | 116/204 | 0.666(0.516~0.861) | **0.002*** |
|  | G | 604/600 | 1 |  | 92/600 | 1 |  | 512/600 | 1 |  |
|  |  |  |  |  |  |  |  |  |  |  |
| **rs4957341** | All |  |  | 0.099 |  |  | **0.003*** |  |  | 0.467 |
|  | AA | 26/14 | 1.849(0.939~3.641) |  | 10/14 | 3.269(1.348~7.927) | **0.003*** | 16/14 | 1.444(0.687~3.037) |  |
|  | AG | 140/158 | 0.908(0.677~1.218) |  | 26/158 | 0.809(0.478~1.370) |  | 114/158 | 0.939(0.687~1.283) |  |
|  | GG | 228/230 | 1 |  | 46/230 | 1 |  | 182/230 | 1 |  |
|  | A | 192/186 | 1.070(0.850~1.348) | 0.564 | 46/186 | 1.295(0.887~1.890) | 0.179 | 146/186 | 1.015(0.792~1.300) | 0.907 |
|  | G | 596/618 | 1 |  | 118/618 | 1 |  | 478/618 | 1 |  |
|  |  |  |  |  |  |  |  |  |  |  |
| **rs4133101** | All |  |  | 0.076 |  |  | 0.076 |  |  | 0.216 |
|  | CC | 100/82 | 1.118(0.751~1.665) |  | 24/82 | 1.279(0.672~2.432) |  | 76/82 | 1.085(0.709~1.660) |  |
|  | CT | 184/220 | 0.776(0.556~1.084) |  | 34/220 | 0.689(0.386~1.229) |  | 150/220 | 0.804(0.564~1.146) |  |
|  | TT | 110/102 | 1 |  | 24/102 | 1 |  | 86/102 | 1 |  |
|  | C | 384/384 | 1.050(0.862~1.277) | 0.630 | 82/384 | 1.104(0.789~1.545) | 0.563 | 302/384 | 1.036(0.840~1.276) | 0.743 |
|  | T | 404/424 | 1 |  | 82/424 | 1 |  | 322/424 | 1 |  |
|  |  |  |  |  |  |  |  |  |  |  |
| **rs4546432** | All |  |  | 0.251 |  |  | 0.096 |  |  | 0.565 |
|  | TT | 96/84 | 1.067(0.716~1.591) |  | 24/84 | 1.251(0.658~2.377) |  | 72/84 | 1.028(0.670~1.577) |  |
|  | CT | 190/218 | 0.822(0.589~1.148) |  | 34/218 | 0.695(0.389~1.240) | 0.027# | 156/218 | 0.863(0.605~1.230) |  |
|  | CC | 108/102 | 1 |  | 24/102 | 1 |  | 84/102 | 1 |  |
|  | T | 382/386 | 1.029(0.845~1.252) | 0.778 | 82/386 | 1.093(0.781~1.530) | 0.603 | 300/386 | 1.012(0.821~1.248) | 0.909 |
|  | C | 406/422 | 1 |  | 82/422 | 1 |  | 324/422 | 1 |  |
|  |  |  |  |  |  |  |  |  |  |  |
| **rs4383756** | All |  |  | 0.958 |  |  | 0.398 |  |  | 0.973 |
|  | GG | 30/32 | 0.966(0.567~1.646) |  | 4/32 | 0.642(0.214~1.922) |  | 26/32 | 1.041(0.597~1.814) |  |
|  | GT | 150/150 | 1.040(0.775~1.397) |  | 36/150 | 1.242(0.757~2.039) |  | 114/150 | 0.983(0.717~1.347) |  |
|  | TT | 214/222 | 1 |  | 42/222 | 1 |  | 172/222 | 1 |  |
|  | G | 210/214 | 1.008(0.808~1.259) | 0.941 | 44/214 | 1.018(0.697~1.487) | 0.927 | 166/214 | 1.006(0.794~1.274) | 0.960 |
|  | T | 578/594 | 1 |  | 120/594 | 1 |  | 458/594 | 1 |  |

SNPs in *PTGER4* are compared between all AS patients, severe AS patients, and normal AS patients versus the control subjects. The rs10440635 SNP shows significant difference when comparing severe AS patients to controls, AA genotype is higher than controls (p=1.126×10^-6^), A allele is higher than controls (p=1.667×10^-6^); this SNP also shows significant difference when comparing normal AS to controls, AA genotype is lower than controls (p=1.763×10^-5^), A allele is lower than controls (p=0.002). The rs4957341 SNP shows significant difference when comparing severe AS to controls, AA genotype is higher than controls (p=0.003).
