# Supplementary material for: JARID1A, JMY, and PTGER4 Polymorphisms Are Related to Ankylosing Spondylitis in Chinese Han Patients: A Case-Control Study
Source: PLoS One. 2013 Sep 19;8(9):e74794. doi: 10.1371/journal.pone.0074794 (PMC3777963; doi:10.1371/journal.pone.0074794)
Supplement: Table S4 — Haplotype analysis comparing all AS patients to controls. Haplotypes are constructed due to LD map (Figure 2). Case ratio means in the case group, the frequency of this kind of haplotype vs. other kinds of haplotype; control ratio means in the control group, the frequency of this kind of haplotype vs. other kinds of haplotype. Block 2 contains rs4133101 rs4546432 and rs4383756 SNPs in PTGER4. CTT frequency is higher than controls (p=6.266×10^-8^). (DOCX) [file pone.0074794.s006.docx]

Table S4 Haplotype analysis comparing all AS patients to controls.

|  | Haplotype | Case ratio | control ratio | OR (95%CI) | p-value |
| --- | --- | --- | --- | --- | --- |
| block 1 | TT | 416:376 | 454:354 | 0.863(0.709~1.050) | 0.141 |
|  | AT | 288:504 | 267:541 | 1.158(0.942~1.423) | 0.163 |
|  | AG | 85:707 | 82:726 | 1.064(0.773~1.467) | 0.703 |
| block 2 | CTT | 382:410 | 282:526 | 1.738(1.422~2.124) | 6.266E-8* |
|  | TCG | 206:586 | 212:596 | 0.988(0.791~1.235) | 0.918 |
|  | TCT | 196:596 | 208:600 | 0.949(0.757~1.189) | 0.647 |
| block 3 | CCA | 370:422 | 361:447 | 1.086(0.892~1.322) | 0.413 |
|  | TTA | 262:530 | 241:567 | 1.163(0.942~1.437) | 0.161 |
|  | CTA | 101:691 | 121:687 | 0.830(0.624~1.103) | 0.198 |
|  | CTG | 56:736 | 81:727 | 0.683(0.478~0.975) | 0.035# |

Haplotypes are constructed due to LD map (Figure 2). Case ratio means in the case group, the frequency of this kind of haplotype vs. other kinds of haplotype; control ratio means in the control group, the frequency of this kind of haplotype vs. other kinds of haplotype. Block 2 contains rs4133101 rs4546432 and rs4383756 SNPs in *PTGER4*. CTT frequency is higher than controls (p=6.266×10^-8^) .
