# Supplementary material for: JARID1A, JMY, and PTGER4 Polymorphisms Are Related to Ankylosing Spondylitis in Chinese Han Patients: A Case-Control Study
Source: PLoS One. 2013 Sep 19;8(9):e74794. doi: 10.1371/journal.pone.0074794 (PMC3777963; doi:10.1371/journal.pone.0074794)
Supplement: Table S5 — Haplotype analysis comparing severe AS patients to controls. Haplotypes are constructed due to Figure A1. Case ratio means in the severe AS group, the frequency of this kind of haplotype vs. other kinds of haplotype; control ratio means in the control group, the frequency of this kind of haplotype vs. other kinds of haplotype. Block 1 contains rs7134353 and rs4980880 SNPs in JARID1A. TT is lower than controls (p=4.136×10^-4^). Block 3 contains rs16876619 and rs4704556 SNPs in JMY. CC is higher than controls (p=2.682×10^-7^). CT is lower than controls (p=4.660×10^-5^). (DOCX) [file pone.0074794.s007.docx]

Table S5 Haplotype analysis comparing severe AS patients to controls.

|  | Haplotype | Case ratio | control ratio | OR (95%CI) | p-value |
| --- | --- | --- | --- | --- | --- |
| block 1 | TT | 67:97 | 452:356 | 0.544(0.387~0.765) | 4.136E-4* |
|  | AT | 77:87 | 266:542 | 1.803(1.284~2.534) | 0.001* |
|  | AG | 20:144 | 81:727 | 1.247(0.740~2.099) | 0.406 |
| block 2 | CTT | 82:82 | 381:427 | 1.121(0.801~1.568) | 0.506 |
|  | TCG | 44:120 | 211:597 | 1.037(0.710~1.516) | 0.849 |
|  | TCT | 38:126 | 208:600 | 0.870(0.586~1.292) | 0.490 |
| block 3 | CC | 110:54 | 364:444 | 2.485(1.744~3.540) | 2.682E-7* |
|  | TT | 38:126 | 243:565 | 0.701(0.474~1.038) | 0.075 |
|  | CT | 16:148 | 195:613 | 0.340(0.198~0.583) | 4.660E-5* |

Haplotypes are constructed due to Figure A1. Case ratio means in the severe AS group, the frequency of this kind of haplotype vs. other kinds of haplotype; control ratio means in the control group, the frequency of this kind of haplotype vs. other kinds of haplotype. Block 1 contains rs7134353 and rs4980880 SNPs in *JARID1A*. TT is lower than controls (p=4.136×10^-4^). Block 3 contains rs16876619 and rs4704556 SNPs in *JMY*. CC is higher than controls (p=2.682×10^-7^). CT is lower than controls (p=4.660×10^-5^).
