# Supplementary material for: JARID1A, JMY, and PTGER4 Polymorphisms Are Related to Ankylosing Spondylitis in Chinese Han Patients: A Case-Control Study
Source: PLoS One. 2013 Sep 19;8(9):e74794. doi: 10.1371/journal.pone.0074794 (PMC3777963; doi:10.1371/journal.pone.0074794)
Supplement: Table S6 — Haplotype analysis comparing severe AS patients to controls. Haplotypes are constructed. Case ratio means in the case group, the frequency of this kind of haplotype vs. other kinds of haplotype; control ratio means in the control group, the frequency of this kind of haplotype vs. other kinds of haplotype. Block 3 contains rs16876619, rs4704556 and rs16876657 SNPs in JMY. TTA is marginal significant higher than controls but cannot pass Bonferroni correction. (DOCX) [file pone.0074794.s008.docx]

Table S6 Haplotype analysis comparing severe AS patients to controls.

|  | Haplotype | Case ratio | control ratio | OR (95%CI) | p-value |
| --- | --- | --- | --- | --- | --- |
| block 1 | TT | 349:279 | 454:354 | 0.975(0.791~1.203) | 0.816 |
|  | AT | 210:418 | 267:541 | 1.018(0.816~1.270) | 0.875 |
|  | AG | 65:563 | 82:726 | 1.022(0.725~1.441) | 0.900 |
| block 2 | CTT | 300:328 | 382:426 | 1.020(0.828~1.257) | 0.853 |
|  | TCG | 162:466 | 212:596 | 0.977(0.771~1.240) | 0.850 |
|  | TCT | 158:470 | 208:600 | 0.970(0.763~1.232) | 0.801 |
| block 3 | CCA | 260:368 | 364:444 | 0.862(0.698~1.064) | 0.167 |
|  | TTA | 224:404 | 244:564 | 1.282(1.027~1.600) | 0.028# |
|  | CTA | 91:537 | 118:690 | 0.991(0.737~1.332) | 0.952 |
|  | CTG | 50:578 | 78:730 | 0.810(0.558~1.174) | 0.264 |

Haplotypes are constructed. Case ratio means in the case group, the frequency of this kind of haplotype vs. other kinds of haplotype; control ratio means in the control group, the frequency of this kind of haplotype vs. other kinds of haplotype. Block 3 contains rs16876619, rs4704556 and rs16876657 SNPs in *JMY*. TTA is marginal significant higher than controls but cannot pass Bonferroni correction.
